# Number-line estimation training in preschoolers supports early mathematical development and is associated with a stronger approximate-to-symbolic numerical pathway

**DOI:** 10.3389/fpsyg.2026.1883264

**Published:** 2026-09-04

**Authors:** Maybí Morell-Ruiz, Eva-Maria Ternblad, Betty Tärning, Sonja Holmer, Magnus Haake, Agneta Gulz

**Affiliations:** Lund University Cognitive Science (LUCS), Lund University, Lund, Sweden

**Keywords:** early math abilities, mapping account, number-line estimation task, refinement account, virtual math game

## Abstract

Understanding how children connect approximate nonsymbolic magnitudes with exact symbolic numbers is a central question in theories of numerical development. A major debate concerns the directionality of this relation: whether approximate magnitude representations scaffold the acquisition of symbolic number knowledge, as proposed by mapping accounts, or whether symbolic number learning refines approximate magnitude processing, as proposed by refinement accounts. This study examined whether number-line estimation training enhances preschoolers’ number-line accuracy and early mathematical abilities, and whether it is associated with short-term developmental pathways between nonsymbolic and symbolic numerical systems. A total of 114 Swedish preschoolers participated in a pre-post intervention study, with an experimental group receiving three sessions of game-like computerized number-line estimation practice on a bounded 0–20 number line with trial-by-trial feedback. Results showed a large improvement in number-line accuracy and a modest but significant transfer effect to early mathematical abilities in the experimental group, whereas the control group showed no comparable gains in these intervention outcomes. Analyses of symbolic and nonsymbolic numerical processing revealed two complementary predictive dynamics. Symbolic abilities predicted later nonsymbolic performance after controlling for baseline nonsymbolic performance and condition, consistent with the refinement account. Conversely, nonsymbolic abilities predicted later symbolic performance after controlling for baseline symbolic performance and condition, consistent with predictions from the mapping account. Importantly, *post-hoc* analyses yielded converging, moderate directional evidence that this approximate-to-symbolic predictive relation was stronger in the experimental group than in the control group, consistent with the possibility that number-line training supports the coordination of approximate and symbolic numerical representations. These findings are consistent with a bidirectional view of numerical development in which symbolic knowledge refines approximate magnitude processing, while approximate numerical abilities can support symbolic development when children engage in learning activities that bridge both systems. By positioning number-line estimation as both an educational tool and a bridge between numerical systems, this study may help clarify mixed findings on developmental directionality and highlights a brief, scalable activity for scaffolding early mathematical learning.

## 1 Introduction

Numerical information is everywhere, not only in school settings but also in the decisions we make in our daily lives. Investigating how children develop their understanding of numerosity, and which tasks can stimulate this development is crucial from both a theoretical and practical perspective. It can contribute to a better understanding of the cognitive mechanisms underlying decision-making that involves numerical information and to guiding educational practices.

Despite extensive research on the interplay between symbolic and approximate numerical systems, a significant theoretical debate persists regarding alternative developmental pathways in the interaction of these systems and how they influence the development of numerical cognition. Additionally, studies on the number line have shown mixed results regarding transfer effects on early math skills, while also suggesting that number line training may be associated with a stronger developmental pathway from approximate to symbolic numerical systems. This study aims to contribute to the literature by employing a controlled intervention to evaluate the direct and transfer effects of number-line training, as well as whether it is associated with differences in the predictive relations corresponding to these developmental pathways. Beyond contributing to this theoretical controversy, understanding these developmental dynamics is critical for improving developmental outcomes. Identifying how early training can serve as a cognitive “bridge” allows for the design of more effective pedagogical tools that support children during the transition to formal mathematics, ultimately helping to close achievement gaps before academic difficulties become entrenched.

To address these aims, the introduction first outlines the theoretical debate concerning the developmental relation between approximate and symbolic numerical systems. It then reviews evidence on number-line estimation and its role in early mathematical learning, including mixed findings on transfer from number-line training to broader mathematical skills. Finally, it considers the possibility that number-line estimation may serve as a bridge between approximate and symbolic numerical representations, leading to the hypotheses tested in the present study.

Within research on developmental numerical cognition, we can distinguish between two complementary models for numerical quantities: a nonsymbolic representation based on an evolutionarily ancient Approximate Numerical System (ANS) and an exact, symbolic representation based on the Symbolic Numerical System (SNS). The ANS enables humans—and even animals—to estimate approximate magnitudes, such as arrays of dots, as demonstrated by Feigenson et al. (2004). In contrast, the SNS facilitates understanding of magnitudes using symbolic codes, allowing us to comprehend that the numerical distance between 1 and 2 is the same as that between 187 and 188 (Szkudlarek and Brannon, 2017). While the distinction between these systems is well-documented, the interaction between them, and the developmental dynamics underlying this interaction, remains a topic of ongoing debate in numerical cognition (Szkudlarek and Brannon, 2017; Qiu et al., 2021; Lau et al., 2021; Libertus, 2019; Odic and Starr, 2018; Szűcs and Myers, 2017).

One proposed developmental dynamic, *the mapping account*, involves growth from ANS to SNS (ANS-to-SNS). Acuity in ANS is considered foundational for numerical cognitive processing and is a strong predictor of mathematical success (Dehaene and Changeux, 1993; Feigenson et al., 2004; Piazza, 2010; Szkudlarek and Brannon, 2017). This claim is supported by correlation studies summarized in three meta-analyses, which report significant correlations between ANS acuity and math abilities (Chen and Li, 2014; Fazio et al., 2014; Schneider et al., 2017). Furthermore, intervention studies where nonsymbolic arithmetic tasks are trained show improvements in symbolic math tasks among adults (Park and Brannon, 2013, 2014) and among first-grade children (Hyde et al., 2014). Collectively, these studies suggest that symbolic numerical representation builds upon the ancient skills of the ANS.

Nevertheless, recent research challenges the mapping account (Matejko and Ansari, 2016; Lyons et al., 2018; Lau et al., 2021), proposing an alternative dynamic, namely that SNS development may drive ANS growth (SNS-to-ANS). In a longitudinal study, Lyons et al. (2018) assessed 539 preschoolers using the Numeracy Screener (Nosworthy et al., 2013), a brief instrument comprising symbolic Arabic-numeral and nonsymbolic dot-array comparison tasks, in which children select the larger numerical quantity. Results indicated that children’s symbolic abilities predicted their nonsymbolic development over the course of an academic year, while no evidence was found for the reverse dynamic (ANS-to-SNS). Similarly, in a study with 622 children, Lau et al. (2021) tested two models: the mapping model, in which ANS abilities predict SNS development, and the refinement model, in which SNS skills drive ANS growth. Their results supported *the refinement account* (Lau et al., 2021), questioning whether approximate abilities supported by the ANS can be considered foundational skills in numerical development. Furthermore, in a multilevel meta-analysis investigating the causal effect of ANS training on symbolic math performance, Qiu et al. (2021) found a small and nonsignificant effect of ANS training on symbolic math task performance.

Taken together, findings from the mapping and refinement accounts indicate that symbolic and nonsymbolic numerical systems are closely related in children’s processing of numerical information. At the same time, these perspectives yield mixed evidence regarding the directionality of their interaction: the mapping account supports a developmental trajectory from ANS to SNS, whereas the refinement account proposes the reverse dynamic, from SNS to ANS. Investigating the developmental dynamics underlying the interaction between these systems may help clarify this theoretical debate while informing educational interventions aimed at supporting the development of mathematical competencies in children.

Our study aims to contribute to the debate on the developmental dynamics underlying the interaction between SNS and ANS by examining these bidirectional processes in a sample of 114 preschoolers using a pre- and post-test design with experimental and control groups. Participants’ ability to compare symbolic and nonsymbolic quantities was assessed with the Numeracy Screener at both time points. We evaluated developmental trajectories from SNS to ANS and from ANS to SNS. Both groups were continuously engaged in their regular preschool instructional activities throughout the study period. The experimental group, however, received three additional sessions of number-line estimation training. This design enables us to capture developmental trajectories that emerge under typical educational exposure—comparable to those documented in longitudinal research—while also testing whether these trajectories are associated with targeted training.

A second line of research relevant to the present study concerns number-line estimation, a task closely linked to children’s mathematical development. The Number Line Estimation Task (NLET) requires participants to approximate the location of a target number on a bounded number line marked only at its endpoints (e.g., 0 and 20). Evidence supports the role of number-line estimation in math learning, as NLET high performance—measured by a decrease in the Percentage of Absolute Error (PAE)—correlates with success in mathematical tasks such as counting, arithmetic skills, and overall school achievement (Ashcraft and Moore, 2012; Bailey et al., 2014; Jordan et al., 2013; Hornung et al., 2014). Young children’s ability to perform on number-line tasks has been shown to be associated with their proficiency in arithmetic (Booth and Siegler, 2008), as well as with standardized achievement scores (Sasanguie et al., 2012) and school grades (Schneider et al., 2009). Furthermore, in a meta-analysis by Schneider et al. (2018), the authors reported that the task is a robust tool for diagnosing and predicting math competencies.

Intervention studies, however, show mixed results (Ramani and Siegler, 2011; Siegler and Ramani, 2009; Maertens et al., 2016; Honoré and Noël, 2016; Mera et al., 2022; Wei et al., 2023; Lunardon et al., 2023). Despite the fact that number-line training has been shown to have a significant impact on estimation abilities (Ramani and Siegler, 2011; Siegler and Ramani, 2009; Ramani and Siegler, 2008; Ramani et al., 2012), findings about the transfer effect from number-line training to broader math abilities remain mixed. On one hand, transfer effects to arithmetic were observed after playing with a linear number board (Ramani and Siegler, 2011; Siegler and Ramani, 2009) and a computerized game—Rescue Calcularis—designed by Kucian et al. (2011). Similarly, Maertens et al. (2016) trained 5-year-old children in number comparison and number-line estimation and found that both types of training improved exact arithmetic performance. Honoré and Noël (2016) also reported improvements in both number-line estimation and arithmetic outcomes. Moreover, Mera et al. (2022) trained preschool children using four digital learning games targeting number-line estimation and other numerical skills. Compared with the control group, children who received the intervention showed significantly greater gains in early mathematical abilities (Mera et al., 2022).

On the other hand, more recent studies have found that training children on number-line estimation improved their estimation accuracy but did not yield significant gains in symbolic arithmetic. For instance, Wei et al. (2023) conducted an intervention study with 96 preschoolers divided into two training groups: number-line and magnitude comparison, and two control groups. They found that number-line training improved participants’ performance in the NLET, but no transfer effect was found from this training to number comparison abilities. Similarly, Lunardon et al. (2023) conducted an intervention study with 183 first-to-third grade children divided into three groups: number-line training, attention training, and control. The authors reported enhanced precision in number-line tasks after training but found no significant transfer effects to arithmetic or number comparison tasks.

Taken together, these findings highlight the effectiveness of number-line training in enhancing number-line estimation abilities, and suggest that further research is needed to determine whether transfer effects occur and under what conditions.

Our study aims to shed light on this topic by testing whether a training version of the NLET can improve number-line estimation abilities and produce a positive, significant impact on early math skills in preschoolers. To this end, a training variant of the NLET—the frog-game task—was developed in a game-like setting, combining estimation on a 0–20 number-line with feedback on children’s responses. The feedback is provided on a trial-by-trial basis, integrated into a child-friendly narrative, and aims at supporting children’s learning in a playful way.

Participants in the experimental group completed 30 trials of the frog-game task once per week for three consecutive weeks, estimating the position of a target number in each trial and receiving feedback on its actual location. Experimental and control groups’ number-line estimation abilities were measured by the PAE, and early math abilities were tested using a math test suitable for 6-year-olds and available in Swedish language: the Ani Banani Math Test (ABMT; Størksen and Mosvold, 2013). Additionally, skills in comparing symbolic and nonsymbolic magnitudes were measured using the Numeracy Screener test. Participants took the tests two times, first during the pre-test session and then during the post-test session.

Beyond its role as an educational tool, number-line estimation may also help explain how approximate and symbolic numerical systems become coordinated during development. While the NLET has been used as a tool to assess symbolic numerical skills in children (e.g., Booth and Siegler, 2008; Lyons and Ansari, 2015), it has also been linked to core ANS acuity (e.g., Halberda and Feigenson, 2008), suggesting that not only SNS but also ANS abilities may be recruited when participants complete the task. A recent study by Gunderson and Hildebrand (2021) explored the relation between symbolic and nonsymbolic numerical abilities, spatial skills, and NLET performance, finding support for the link between the number-line task and both SNS and ANS. They studied 612 children (ages 4–10) over 2 years in a longitudinal design with four measurement time points. The results revealed that participants’ initial number-line estimation skills were significantly related to both exact and approximate calculation abilities, even after accounting for children’s spatial skills. Together, these studies indicate that the NLET bridges both numerical systems.

Moreover, results by Wong et al. (2016) indicate that this task may play a role in enhancing the dynamic interaction between these systems in a particular direction from ANS to SNS. They found that NLET accuracy fully mediated the longitudinal relation between children’s ANS acuity and their symbolic arithmetic skills, suggesting that the task may engage processes through which nonsymbolic representations support symbolic numerical development (Wong et al., 2016). However, although findings suggested a directional mediation, the study did not test this relation in an intervention design; therefore, it remains unclear whether training in NLET may be associated with a stronger developmental pathway from ANS to SNS.

Taken together, findings on the link between the NLET and numerical systems (e.g., Booth and Siegler, 2008; Lyons and Ansari, 2015; Halberda and Feigenson, 2008; Gunderson and Hildebrand, 2021), along with results by Wong et al. (2016) on the mediating role of NLET performance in the relation between ANS and SNS skills, suggest that the NLET integrates the ability to approximate quantities with the capacity to manipulate exact symbolic magnitudes. Moreover, findings open the question of whether training targeting number-line abilities might enhance the developmental pathway from ANS to SNS.

In the current study, we used a pretest–posttest design with experimental and control groups, in which participants in the experimental group received training in number-line estimation abilities through the frog-game task. Experimental and control groups followed their regular preschool curriculum. By employing this approach, we aim to examine developmental relations between symbolic and nonsymbolic numerical abilities, such as mapping and refinement pathways, under two learning contexts: regular preschool instruction and additional number-line estimation training.

Finally, building upon prior findings, the present study has two primary purposes. First (Objective 1), it aims to investigate the effects of number-line training on number-line estimation abilities (direct effect) and early math abilities (transfer effect) in preschool children, providing evidence that may help clarify the mixed findings on number-line transfer effects reported in previous research. Second (Objective 2), the study examines developmental pathways in the interaction between the SNS and the ANS under two learning contexts (regular preschool instruction and number-line estimation training), with the goal of shedding light on the theoretical debate regarding the directionality of their interaction.

Regarding the direct training effect, we expect that participants in the experimental group who receive number-line estimation training will show greater improvements in number-line estimation accuracy than those in the control group. Our first hypothesis (H1) is therefore that *number-line estimation skills will improve more from pre-test to post-test in the experimental group than in the control group, as indicated by a greater reduction in the Percentage of Absolute Error (PAE)*.

Regarding the transfer effect, although the literature reports mixed findings, some studies suggest that number-line training in preschoolers can enhance broader mathematical competencies under specific conditions (Ramani and Siegler, 2011; Siegler and Ramani, 2009; Maertens et al., 2016; Honoré and Noël, 2016; Mera et al., 2022). Accordingly, our second hypothesis (H2) is that *early math abilities, as measured by the ABMT, will show greater improvement from pre-test to post-test in the experimental group compared to the control group*.

Concerning the second purpose of the study, we examined the developmental dynamics underlying the interaction between the ANS and the SNS by testing predictions derived from the refinement and mapping accounts. Based on longitudinal evidence in preschool children (Lyons et al., 2018), the refinement account proposes that symbolic numerical competence supports the development of nonsymbolic magnitude processing. Accordingly, the first part of our third hypothesis (H3a) is that *pre-test symbolic abilities will predict post-test nonsymbolic skills after controlling for baseline nonsymbolic performance and condition.* This would be consistent with the refinement account proposing that symbolic knowledge supports the development of approximate magnitude representations, aligning with longitudinal findings on the spontaneous emergence of the pathway from SNS to ANS (Matejko and Ansari, 2016; Lyons et al., 2018; Lau et al., 2021; Mou et al., 2023).

The mapping account proposes growth from nonsymbolic to symbolic representations, although recent findings in children have been mixed (Matejko and Ansari, 2016; Hyde et al., 2014; Lyons et al., 2018; Szkudlarek and Brannon, 2018). Given evidence suggesting that number-line estimation integrates approximate and symbolic representations (Gunderson and Hildebrand, 2021; Wong et al., 2016), we expect that training may be associated with a stronger predictive relation along this pathway. Thus, the second part of our third hypothesis (H3b) is that *the experimental and control groups will show different developmental patterns in the progression from ANS to SNS. Specifically, we expect pre-test nonsymbolic abilities to predict post-test symbolic skills in the experimental group but not in the control group, suggesting that number-line training may be associated with a stronger pathway from nonsymbolic to symbolic representations*.

In summary, this study examines the effects of a number-line estimation intervention on preschoolers’ number-line accuracy and early math skills by comparing pre- and post-test performance between experimental and control groups. Additionally, it investigates the directionality of the relation between the ANS and SNS by testing two well-documented developmental pathways: the progression from ANS to SNS proposed by the mapping account and the reverse pathway from SNS to ANS proposed by the refinement account. In both cases, we examine whether number-line training is associated with these developmental dynamics.

By testing alternative developmental pathways in the interaction between symbolic and nonsymbolic numerical systems, this study aims to contribute to the theoretical debate regarding foundational mechanisms in numerical development. In addition, beyond examining the developmental dynamics between the ANS and SNS proposed by the mapping and refinement accounts, the present study is, to our knowledge, among the first to investigate whether a brief and scalable number-line training intervention may shape, or be associated with, these short-term developmental relations in preschool children. By integrating perspectives from cognitive science, numerical cognition, and developmental and educational psychology, we aim to connect well-established theoretical frameworks with practical approaches for fostering numerical development in early childhood.

## 2 Materials and methods

### 2.1 Participants

The study was conducted in three schools in the municipality of Lund and neighboring municipalities in southern Sweden, involving six preschool classes. All three schools agreed to participate after being contacted via email and informed that the study aimed to enhance preschoolers’ math skills through NLET training. All schools were located in areas with medium-to-high socioeconomic status.

Overall, the study involved a total of 114 children, including 55 girls and 59 boys. In accordance with the approved ethical protocol and its data-minimization requirements, information that could allow individual children to be identified was not collected. Consequently, participants’ date of birth was not collected. Ages at the start of the study ranged from 5;9 to 6;9.

Assignment to condition was conducted primarily at the classroom level, following a quasi-experimental design. Five classes were randomly assigned to either the experimental condition or the control condition: classes 1, 2, and 4 were assigned to the experimental group, and classes 3 and 5 were assigned to the control group. To avoid imbalance in group sizes, the sixth class was randomly divided between conditions at the individual-child level, with 11 children assigned to the experimental group and 6 children assigned to the control group. Classroom-level randomization was used, where possible, to preserve the natural classroom context, minimize disruption to ordinary preschool routines, and reduce the risk of children feeling excluded from the game-based training. This resulted in a total of 58 participants in the experimental group (30 girls, 28 boys) and 56 participants in the control group (25 girls, 31 boys). The sex distribution did not differ significantly between groups, as indicated by the chi-square test *χ*^2^(1) = 0.572, *p* = 0.449, Cramér’s *V* = 0.07.

All participants were enrolled in regular preschool programs during the study period. Participants in the control group were offered the training after the post-test assessment, ensuring equal beneficial learning opportunities for all participants involved in the study, as recommended by the ethical guidelines in the Swedish educational context.

Of the total number of students enrolled in preschool classes at the participating schools, almost all participated in this study. Only 29 students were excluded because they were enrolled in an alternative educational intervention program, which is part of a larger study on virtual versus physical interaction and its impact on children’s math competencies.

### 2.2 Procedure

Data collection occurred in fall 2023 and included two individual testing sessions (pre- and post-test) for all participants, and three training sessions for the experimental group.

Testing took place in quiet rooms at the schools, with each child completing each session on a one-to-one basis. Each session lasted approximately 10–15 min, and the time window between pre- and post-test sessions ranged from 5 to 7 weeks. Experimenters assessed participants’ number-line abilities with the NLET, early math skills with the ABMT, and symbolic and nonsymbolic magnitude comparison skills via the Numeracy Screener. To control for order effects, both the sequence of instrument administration and the assignment of experimenters were counterbalanced.

In addition to the testing sessions, the experimental group also received three 10-min training sessions using the frog-game task. Training sessions took place once a week during three consecutive weeks. The control group did not receive additional training during this period, beyond their regular preschool activities.

### 2.3 Measurements and frog-game task

The current study involved the assessment of number-line estimation (Section 2.3.1), early math abilities (Section 2.3.2), and symbolic and nonsymbolic skills (Section 2.3.3) in preschoolers. Participants were assessed at both pre-test and post-test. Additionally, children in the experimental group completed three training sessions using the frog-game number-line task (Section 2.3.4).

#### 2.3.1 Number-line estimation abilities

Number-line estimation abilities were assessed using the Number Line Estimation Task (NLET; Siegler and Booth, 2004), which evaluates a participant’s ability to estimate numerical values on an empty number-line labeled with a starting point (e.g., 0) and an endpoint (e.g., 20).

The task was conducted on iPads, and participants provided their answers individually by touching the line on the screen. The experimenter accompanied each child throughout the task, without providing feedback. The task began with audio instruction, followed by three practice trials during which children had the opportunity to ask questions before the testing phase started.

**Figure 1:**
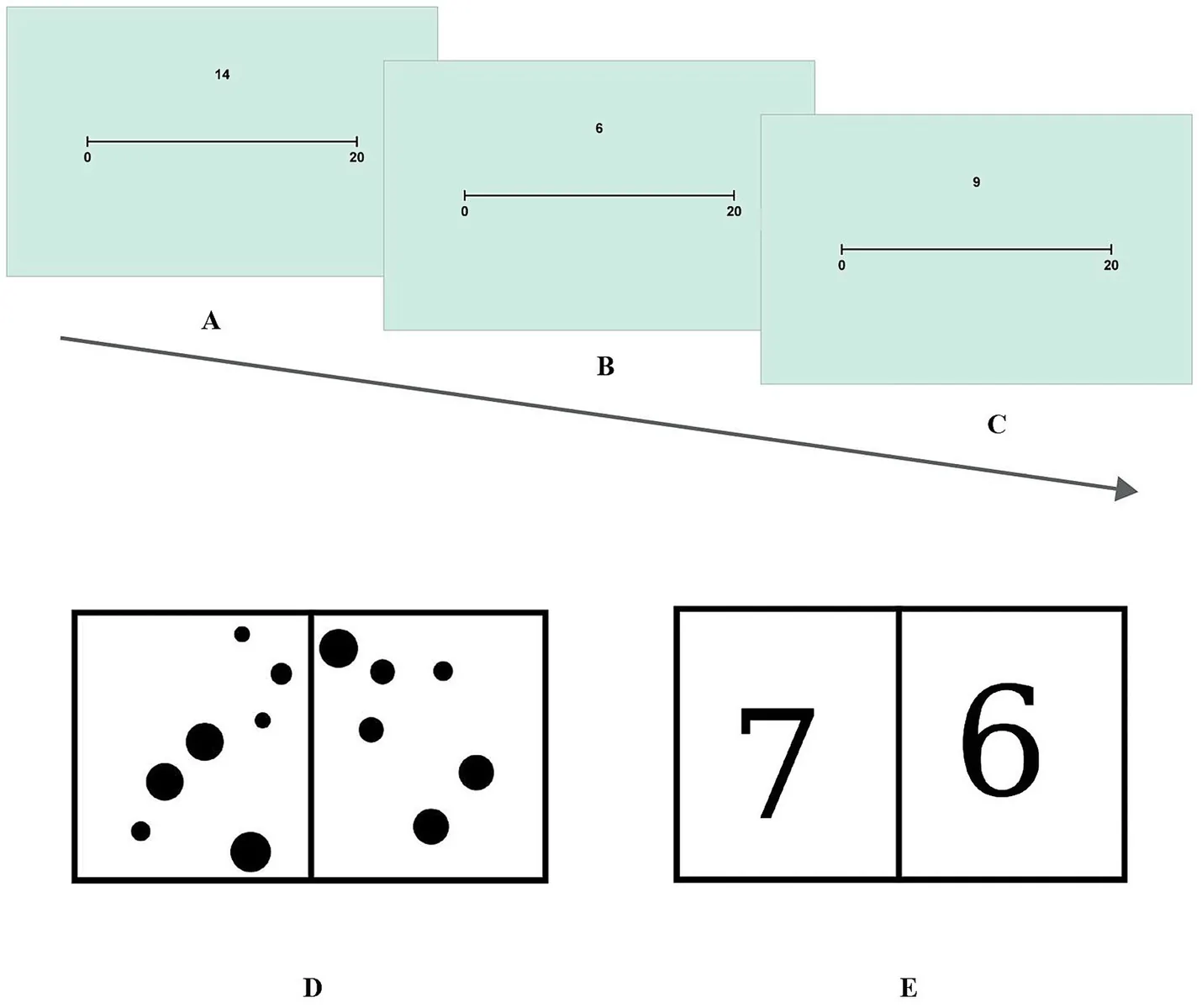
Three trial examples **(A–C)** in the Number Line Estimation Task (NLET) and two examples **(D,E)** of nonsymbolic and symbolic trials from the Numeracy Screener. For the NLET, targets 14 **(A)**, 6 **(B)** and 9 **(C)** are shown. For the Numeracy Screener, panel **(D)** shows a nonsymbolic trial (NST), and panel **(E)** shows a symbolic trial (ST).

Figures 1A–C illustrate three example trials in the testing phase. The number-line ranges from 0 to 20 and is placed in the center of the screen. The target number appears above the line, as shown in Figure 1, with examples of 14 in A, 6 in B, and 9 in C.

Each participant estimated one target number per trial and completed 15 trials per session in both the pre-test and post-test. The list of targets included 15 random numbers from 1 to 19, presented in random order while controlling for repetitions. No time limit was set for each trial. The task lasted approximately 5 min per participant.

Participant performance in the NLET was scored following the methodology outlined by Siegler and Booth in their seminal 2004 study. Percent Absolute Error (PAE) was used as the primary metric to quantify estimation accuracy, calculated as PAE = 100 × |(Estimate – Estimated Quantity)|/(Scale of Estimate), where Estimate represents the value estimated by the child (indicated by the click location on the line), Estimated Quantity is the actual target number, and Scale of Estimate refers to the range of the number-line (e.g., 20 for a line ranging from 0 to 20).

In the present sample, internal consistency across the 15 assessment trials was high at both pre-test, Cronbach’s *α* = 0.874, and post-test, Cronbach’s *α* = 0.893.

#### 2.3.2 Early math abilities

The participants’ early math abilities were assessed using the Ani Banani Math Test (ABMT), a tool developed by Størksen and Mosvold (2013) and suitable for testing early math abilities in 6-year-olds. Originally designed in Norway, the ABMT is available in both Norwegian and Swedish. This digital test adopts a game-based format and consists of 18 items that evaluate arithmetic, geometry, and problem-solving skills. In the test, children assist a monkey in completing various tasks, such as counting bananas and organizing a table for a party.

The experimenter started the testing process by introducing Ani Banani and proceeded with trials in a fixed order. Participants provided their answers verbally, and the experimenter recorded them directly on the iPad. Task performance is measured by the total number of correct answers.

For this study, three ABMT items addressing problem-solving with graphical patterns were excluded: one item to complete a puzzle (a drawing of a child’s face) and two items to copy simple bead plate patterns. The rationale for excluding these three items was aimed to shorten the total testing time to minimize any disruption to the children’s school activities. Based on our previous experiences with ABMT (Gulz and Haake, 2021), these items tend to be particularly time-consuming for many children, and their inclusion could result in significant delays. To further support the exclusion, a reliability analysis using Cronbach’s alpha was conducted on the remaining items. Internal consistency of the 15 retained ABMT items was acceptable in the present sample at both pre-test, Cronbach’s *α* = 0.715, and post-test, Cronbach’s *α* = 0.758, suggesting that the exclusion of these items did not compromise the overall reliability of the ABMT.

#### 2.3.3 Numeracy Screener

The Numeracy Screener is a paper-based test developed by the Laboratory for Numerical Cognition at the University of Western Ontario (Nosworthy et al., 2013). It is designed to assess children’s ability to compare nonsymbolic and symbolic magnitudes by presenting pairs of quantities and asking participants to identify the larger one. The task consists of two blocks of 56 trials each, yielding a total of 56 nonsymbolic trials (NST) and 56 symbolic trials (ST). Within each block, participants completed as many trials as possible within a one-minute time limit.

Two versions of the test were administered: Form A (NST followed by ST) and Form B (reverse order). Participants were evenly distributed across the two forms. Before each block, the experimenter explained the task and provided practice trials to ensure understanding.

In each nonsymbolic trial, participants chose between two sets of dots. As described by Nosworthy et al. (2013), in half of the trials, the two sets are matched on cumulative surface area, whereas in the other half, they are matched on total perimeter. The two types of trials are presented in mixed order. Participants were instructed not to count the dots.

In each symbolic trial, they compared two Arabic numerals. Figures 1D,E illustrate examples of NST and ST, respectively. Figure 1D (NST) shows a set of six dots and a set of seven dots, whereas Figure 1E (ST) presents a comparison between the numerals 6 and 7. Participants responded by drawing a line with a pencil over the set containing seven dots in Figure 1D and over the numeral 7 in Figure 1E.

Total correct and incorrect answers in NST and ST were calculated across trials. Scoring followed Lyons et al. (2018), adjusting for guessing using the formula *A* = *C* – *I*/(*P* – 1), where *C* = correct, *I* = incorrect, and *p* = 2 (forced-choice), resulting in four individual adjusted scores per participant: two for NST (NST_pre and NST_post) and two for ST (ST_pre and ST_post).

Of the total number of participants, 3 did not complete the Numeracy Screener, and 13 were excluded because they were absent or produced incomplete scores at either the pre-test (5 missing) or post-test (8 missing) session. Incomplete data corresponded to 16 participants in total, 12 of them in the experimental group and 4 in the control group. To evaluate whether excluded participants differed systematically from retained participants, we compared the baseline scores of the eight participants who completed the pre-test but not the post-test with those of participants who completed both sessions. The groups did not differ significantly in either symbolic pre-test scores, Welch’s *t* = −0.58, *p* = 0.577, or nonsymbolic pre-test scores, Welch’s *t* = −0.43, *p* = 0.679. These findings indicate that among participants for whom baseline Numeracy Screener data were available, subsequent exclusion was not associated with systematically lower or higher baseline symbolic or nonsymbolic performance.

The regression analysis was therefore conducted with the 98 participants who were tested in both sessions on symbolic and nonsymbolic abilities. Of these, 46 belonged to the experimental group and 52 to the control group. Descriptive statistics for symbolic and nonsymbolic numerical abilities across condition groups (experimental and control) and sessions (pre-test and post-test) are summarized in Table 1. In the experimental group, 26 participants were girls (56.5%) and 20 were boys (43.5%), whereas in the control group, 25 participants were girls (48.1%) and 27 were boys (51.9%). The sex distribution did not differ significantly between groups, as indicated by the chi-square test *χ*^2^(1) = 0.697, *p* = 0.404, Cramér’s *V* = 0.08.

**Table 1:** Descriptive statistics for symbolic and nonsymbolic numerical abilities by condition group and session, *N* = 98.

Condition groups	Numerical ability	Pre-test *M* (*SE*)	Post-test *M* (*SE*)
Experimental (*N* = 46)	Symbolic	24.28 (1.06)	30.40 (0.88)
	Nonsymbolic	26.25 (0.96)	30.36 (0.68)
Control (*N* = 52)	Symbolic	25.42 (1.28)	27.92 (1.41)
	Nonsymbolic	25.58 (1.03)	30.08 (0.97)

Symbolic = symbolic numerical abilities (SNS); nonsymbolic = approximate number system abilities (ANS). SE, standard error.

As a sample-specific psychometric indicator, the pre–post stability of the guessing-adjusted Numeracy Screener scores was examined in the control group, avoiding the potential influence of intervention-related change on the stability estimates. The correlations indicated moderate-to-high stability for both the symbolic subscale (Pearson’s *r* = 0.869, *n* = 52) and the nonsymbolic subscale (*r* = 0.819, *n* = 52).

#### 2.3.4 Intervention: the frog-game

Based on the NLET, we developed a virtual training activity aimed at supporting children’s numerical estimation skills. In the frog-game, a green frog searches for food along a number-line, advancing with each correct estimate to prepare for a party.

The game was developed in PsychoPy (version 2022.2.5) and deployed online via Pavlovia.^1^ It was optimized for touchscreen interaction and ran on an iPad (screen 10.9 inches). Participants provided answers individually by placing their fingers on the line. Each participant completed 30 trials per training session, conducted once per week over three consecutive weeks. In each trial, participants estimated a target number on a 0–20 number-line and received feedback based on their estimation accuracy. A data file per participant was automatically stored at the end of each training session.

The game begins with a screen displaying a 0–20 number-line, where each whole number is labeled with a symbolic digit below its tick mark, while the frog introduces itself and explains the task:

*“I am Göran the frog. I am out looking for food and the food should be somewhere along this line. Can you help me find it?”*

**Figure 2:**
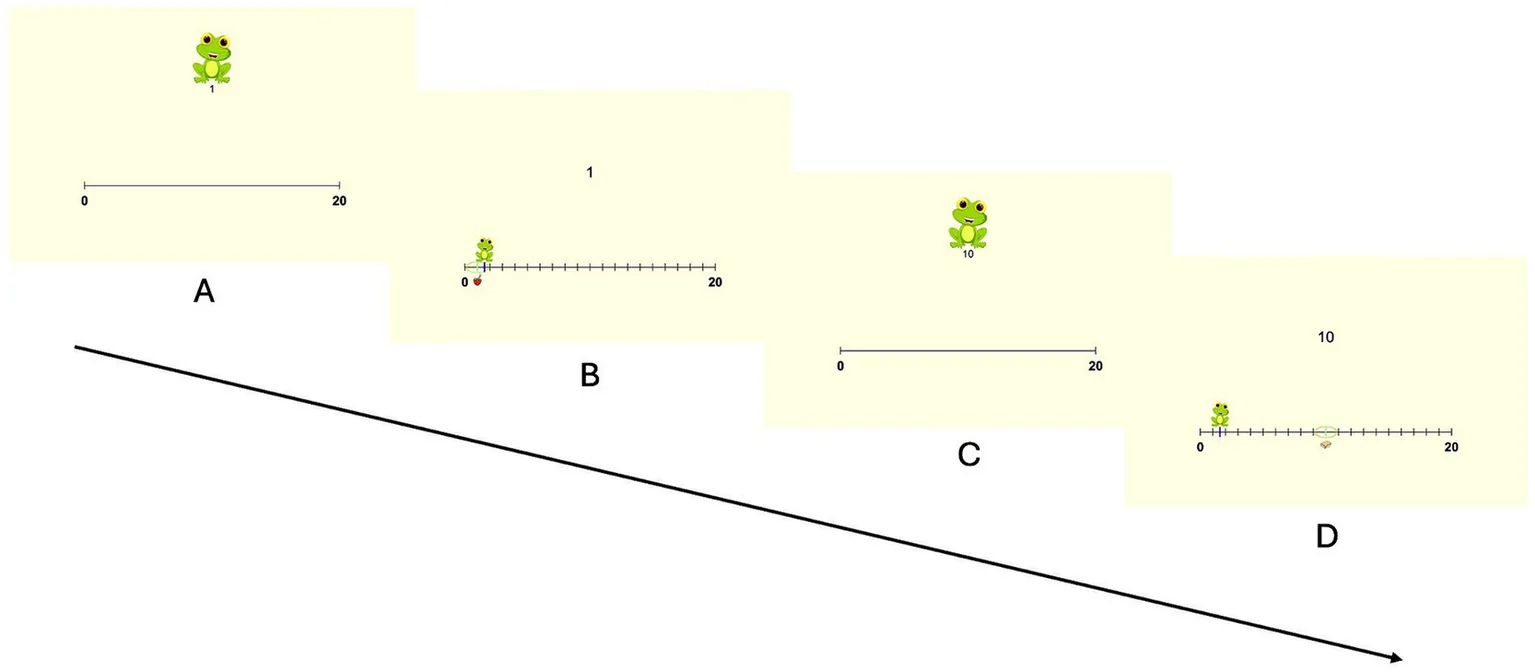
Example trials from the frog-game number-line training task. Panels **(A,C)** show the estimation phase for target numbers 1 and 10, respectively, in which children estimated the target number’s position on a bounded 0–20 number line marked only at the endpoints. Panels **(B,D)** show the corresponding feedback phase, in which tick marks were displayed at each integer. The blue tick indicates the child’s selected location, the food icon marks the exact target location, and the green circle indicates the response zone considered accurate (PAE ≤ 10%). Panel **(B)** illustrates an accurate estimate, in which the selected location falls within the accurate zone, whereas panel **(D)** illustrates an inaccurate estimate, in which the selected location falls outside this zone.

Thus, participants were encouraged to interact with a 0–20 bounded number-line and estimate the location of the target number. Figure 2 illustrates two trial examples, with target numbers 1 (Figures 2A,B) and 10 (Figures 2C,D). During the estimation phase (Figures 2A,C), the line remains unnumbered, with only the endpoints marked. In the feedback phase (Figures 2B,D), tick marks are shown at each whole number location.

After each attempt, children received feedback based on estimation accuracy, with an estimation within a PAE of ≤10% considered correct, and anything outside this range considered incorrect. Figure 2B shows an example of accurate estimation of the location of number one, while Figure 2D illustrates an example of inaccurate estimation of the location of number 10. Participants’ estimations were marked with a blue tick, while the green circle denotes the accurate zone, offering visual cues for self-assessment of performance accuracy. Additionally, a 1-s delayed auditory message confirmed performance accuracy, with positive reinforcement (e.g., “*Pizza! Yummy!*”) if the PAE was ≤10%, or constructive feedback (e.g., *“Oh! The pizza is over there!”*) if the PAE exceeded 10%, to guide improvement.

To maintain engagement, the virtual character changed every 10 trials. Three animal characters were used across three consecutive blocks in the task: the frog, the kangaroo, and the rabbit. Instructions were repeated at the start of each new block, including the presentation of the full numbered line, but with a kangaroo in the second block and a rabbit in the last one. The characters had different voices and used somewhat different wordings to provide variation, while the narrative remained consistent.

The order of target numbers was fixed across blocks. The target number sequence started with 1, 10, 2, 5, 15, 14, 19, 11, 8, and 17 in the frog block; followed by 10, 3, 4, 19, 18, 12, 13, 5, 6, and 7 in the kangaroo block; and ending with 10, 9, 8, 1, 5, 4, 15, 16, 19, and 1 in the rabbit block. This sequence was specially designed to enhance procedural knowledge and encourage the use of various strategies, such as counting up from the starting point (e.g., target numbers 1, 2, 3) and counting down from the upper endpoint (e.g., target numbers 19, 18, 17). The number 10 was overrepresented in the target list, encouraging the use of the midpoint as a landmark for applying estimation strategies based on proportional judgment.

### 2.4 Analysis

The following analyses were conducted to evaluate the study hypotheses and to guide the interpretation of the results. In line with the first objective of the study, we examined whether number-line training improved number-line estimation accuracy and produced transfer effects on early math abilities. We then addressed the second objective by examining the developmental dynamics underlying the interaction between symbolic and nonsymbolic numerical representations and examining whether the intervention condition was associated with differences in predictive relations. To test the direct effect of training on estimation accuracy (H1), Percentage of Absolute Error (PAE) scores from the NLET were analyzed across pre- and post-test sessions for experimental and control groups (*N* = 114). A mixed-model ANOVA was initially conducted with Session (pre-test vs. post-test) as a within-subject factor and Condition (control group vs. experimental group) as a between-subject factor. Residual diagnostics indicated violations of normality assumptions that persisted after logarithmic transformation.

The final analyses were conducted using Generalized Estimating Equations (GEE) with a Gamma distribution and log link because our hypotheses concerned average group-level change from pre-test to post-test rather than individual differences in developmental trajectories. This approach estimates population-averaged effects while accounting for the non-independence of repeated observations within children. GEE was also appropriate given the non-normal residuals observed in the corresponding repeated-measures ANOVA framework and allowed the inclusion of all available outcome observations, including children with data from only one measurement occasion. The primary test of the training effect was the Session × Condition interaction. A significant interaction was interpreted as evidence that the pre- to post-test change differed between the experimental and control groups. To clarify the within-group pattern underlying significant interactions, follow-up model-based simple contrasts were derived from the same GEE model. These contrasts compared pre- and post-test scores within each condition using the model coefficients and robust covariance matrix, and are reported as exponentiated coefficients, *exp*(*B*), corresponding to post/pre ratios under the log link.

To examine whether training effects transferred to broader early math abilities (H2), the same H1 analytic procedure was applied to Ani Banani Math Test (ABMT) scores. A significant Session × Condition interaction was interpreted as evidence that pre- to post-test change in early math abilities differed between the experimental and control groups.

To investigate the developmental relations between symbolic and nonsymbolic magnitude representations (H3), we examined two complementary pathways, as predicted by the refinement and mapping accounts. First, to evaluate the pathway from symbolic to nonsymbolic abilities (H3a), a linear regression analysis was conducted with post-test nonsymbolic abilities as the dependent variable and pre-test symbolic abilities as the predictor. The model controlled for condition and pre-test nonsymbolic abilities, using the following formula:


$$NS_{\mathrm{pos}}\sim NS_{\mathrm{pre}}+S_{\mathrm{pre}}+\text{Condition}$$

where $NS_{\text{post}}$ represents post-test nonsymbolic abilities, $NS_{\mathrm{pre}}$ represents pre-test nonsymbolic abilities, $S_{\mathrm{pre}}$ represents pre-test symbolic abilities and Condition represents group membership (experimental or control). The inclusion of pre-test nonsymbolic abilities as a control follows similar approaches used in longitudinal studies of numerical development (e.g., Lyons et al., 2018; Hutchison et al., 2020). Condition was included in the model, aiming to test adjusted group differences in post-test performance.

Second, to evaluate the pathway from nonsymbolic to symbolic abilities (H3b), a similar linear regression analysis was conducted. In this case, the model included post-test symbolic abilities ( $S_{\text{post}}$ ) as the dependent variable, with pre-test nonsymbolic abilities ( $NS_{\mathrm{pre}}$ ) as predictors. As in the previous model, pre-test symbolic abilities ( $S_{\mathrm{pre}}$ ) and Condition were included, as depicted in the following formula:


$$S_{\text{post}}\sim NS_{\mathrm{pre}}+S_{\mathrm{pre}}+\text{Condition}$$

In the first model, a significant result in the prediction from pre-test symbolic abilities to post-test nonsymbolic abilities—while controlling for condition and pre-test nonsymbolic skills—was interpreted as evidence in support of the predictions proposed by the refinement account. Similarly, in the second model, a significant result in the prediction from pre-test nonsymbolic abilities to post-test symbolic abilities—while controlling for condition and pre-test symbolic skills—was interpreted as evidence in support of the predictions proposed by the mapping account.

In these models, condition effects were interpreted as adjusted group differences in the respective post-test outcome. When significant, it was followed by *post-hoc* analyses. In H3b, to formally test whether the developmental pathway from nonsymbolic pre-test to symbolic post-test performance $\left(\Delta_{\mathrm{NS}}=\beta_{\mathrm{NS},\text{experimental}}-\beta_{\mathrm{NS},\text{control}}\right)$ differed significantly between conditions, we conducted a full-sample interaction analysis using ANCOVA with interactions. The expanded regression model used the following formula:


$$\begin{array}{l} S_{\mathrm{pos}t_{z}}\sim NS_{\mathrm{pr}e_{z}}+S_{\mathrm{pr}e_{z}}+\text{Condition}+\\ S_{\mathrm{pr}e_{z}}\times \text{Condition}+NS_{\mathrm{pr}e_{z}}\times \text{Condition} \end{array}$$

The critical interaction term ( $NS_{\mathrm{pr}e_{z}}\times \text{Condition}$ ) was analyzed using a combined approach, including a frequentist test with this ANCOVA model, and a Bayesian prior-sensitivity analysis to evaluate the directional evidence in the slope difference between groups. In the Bayesian model, the direction in the interaction was measured by $\Delta_{\mathrm{NS}}$ representing the difference between the nonsymbolic-to-symbolic predictive slope in the experimental group ( $\beta_{\mathrm{NS},\text{experimental}}$ ) and the control group ( $\beta_{\mathrm{NS},\text{control}}$ ). Because of the directional nature of the prediction, positive values of the focal parameter $\left(\Delta_{\mathrm{NS}}>0\right)$ indicate a stronger nonsymbolic-to-symbolic relation in the experimental than the control group. The model details are described in Supplementary material 1. *Post-hoc* models were conducted separately for the experimental and control groups to quantify and illustrate the nonsymbolic-to-symbolic predictive relation within each condition.

Finally, we conducted exploratory sensitivity analyses including sex as a covariate to address the potential influence of this variable on the effects and predictive relations examined in the study. Because sex was not part of the theory-driven hypotheses presented in this study, these analyses were used to evaluate the robustness of the main findings and are reported in Supplementary material 2.

## 3 Results

### 3.1 Estimation abilities: Number Line Estimation Task (NLET)

Our first hypothesis (H1) proposes that number-line estimation skills would show greater improvement from pre- to post-test in the experimental group compared to the control group, as measured by a larger decrease in the Percentage of Absolute Error (PAE). To evaluate this, data from all participants (*N* = 114) were analyzed using Generalized Estimating Equations (GEE) with a Gamma distribution and log link function. Results showed a significant interaction effect between Session and Condition (*B* = −0.39; *p* = 0.032), indicating that the experimental group significantly reduced the percentage of absolute error (PAE) from pre- to post-test more than the control group, supporting H1. As an index of effect magnitude, exponentiating the coefficient yielded a relative change ratio of 0.68 (*exp*(*B*) = 0.68, 95% CI [0.48, 0.96]), indicating a meaningful relative effect, corresponding to an approximately 32% greater reduction in PAE in the experimental group than in the control group.

**Figure 3:**
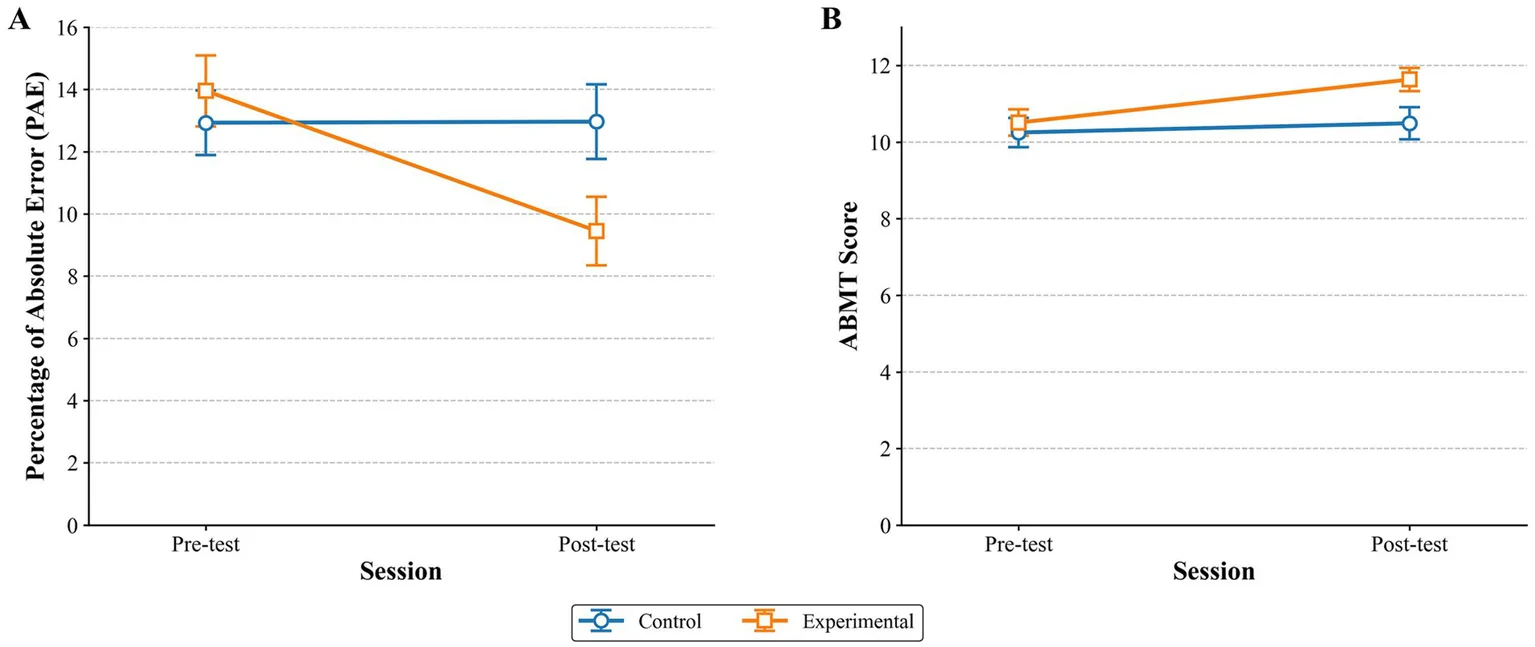
Intervention effects across pre-test and post-test sessions for the control and experimental groups. **(A)** Percentage of absolute error (PAE) in the Number Line Estimation Task. Lower PAE values indicate higher estimation accuracy. **(B)** Ani Banani Math Test (ABMT) scores. Higher scores indicate better early mathematical performance. In both panels, the control group is shown in blue with circular markers, and the experimental group is shown in orange with square markers. Error bars represent standard errors (SE).

These findings are illustrated in Figure 3A, which shows the progression of Percentage of Absolute Error (PAE) across pre- and post-test sessions for the experimental (orange with square markers) and control (blue with circular markers) groups. The control group exhibited stable number-line estimation performance across sessions, as indicated by PAE scores at pre-test (*M* = 12.93, *SE* = 1.04) and post-test (*M* = 12.96, *SE* = 1.19). A follow-up model-based simple contrast derived from the same GEE model confirmed that this within-group change was not statistically significant, *exp*(*B*) = 1.003, 95% CI [0.815, 1.233], Holm-adjusted *p* = 0.980. In contrast, the experimental group showed a reduction in PAE scores from pre-test (*M* = 13.95, *SE* = 1.14) to post-test (*M* = 9.45, *SE* = 1.18). The corresponding model-based simple contrast indicated that this within-group reduction was statistically significant, *exp*(*B*) = 0.677, 95% CI [0.506, 0.907], Holm-adjusted *p* = 0.018, corresponding to an approximately 32.3% reduction in PAE.

Together, the significant Session × Condition interaction and the follow-up model-based simple contrasts with Holm-adjusted *p*-values indicate that the improvement in number-line estimation accuracy was specific to the experimental group. Whereas the control group remained stable across sessions, the experimental group showed a significant reduction in PAE from pre- to post-test, supporting H1.

### 3.2 Early math abilities: the Ani Banani Math Test (ABMT)

Our second hypothesis (H2) was that early math abilities would show greater improvement from pre- to post-test in the experimental group compared to the control group, as measured by a larger increase in the Ani Banani Math Test scores. To test this, data from all participants (*N* = 114) were analyzed using Generalized Estimating Equations (GEE) with a Gamma distribution and log link function. Results indicated a significant interaction effect between Session and Condition (*B* = 0.08; *p* = 0.021), indicating that ABMT scores improved from pre- to post-test in the experimental group more than in the control group. As an index of effect magnitude, exponentiating the coefficient yielded a relative change ratio of 1.08 (*exp*(*B*) = 1.08, 95% CI [1.01, 1.16]), indicating a modest but meaningful relative effect, corresponding to an approximately 8% greater improvement in ABMT scores in the experimental group than in the control group.

These results are illustrated in Figure 3B, which shows the progression of ABMT performance from pre- to post-test sessions for the experimental (orange with square markers) and control (blue with circular markers) groups. The control group showed stable early math abilities across sessions, as indicated by ABMT scores from pre-test (*M* = 10.25, *SE* = 0.38) to post-test (*M* = 10.49, *SE* = 0.42). A follow-up model-based simple contrast derived from the same GEE model confirmed that this within-group change was not statistically significant, *exp*(*B*) = 1.024, 95% CI [0.976, 1.073], Holm-adjusted *p* = 0.337. In contrast, the experimental group showed an increase in ABMT scores from pre-test (*M* = 10.51, *SE* = 0.34) to post-test (*M* = 11.63, *SE* = 0.32). The corresponding model-based simple contrast indicated that this within-group increase was statistically significant, *exp*(*B*) = 1.107, 95% CI [1.055, 1.161], Holm-adjusted *p* < 0.001, corresponding to an approximately 10.7% increase in ABMT scores.

Together, the significant Session × Condition interaction and the follow-up model-based simple contrasts with Holm-adjusted *p*-values indicate that the improvement in early math abilities was specific to the experimental group. Whereas the control group remained stable across sessions, the experimental group showed a significant increase in ABMT scores from pre- to post-test, supporting H2.

### 3.3 Symbolic and nonsymbolic magnitude representations: Numeracy Screener

To examine the developmental dynamics between ANS and SNS, we tested H3 by examining the directionality in the relation between ANS and SNS. We examined refinement and mapping pathways: SNS-to-ANS (H3a) in subsection 3.3.1 and ANS-to-SNS (H3b) in subsection 3.3.2. Finally, in subsection 3.3.3, we present *post-hoc* analyses related to H3b, examining whether the intervention was associated with differences in the ANS-to-SNS predictive relation proposed by the mapping account.

#### 3.3.1 Refinement pathway

Our H3a proposes that pre-test symbolic abilities would predict post-test nonsymbolic skills after controlling for baseline nonsymbolic performance and condition. To test this, a linear regression model examined whether pre-test symbolic abilities predicted post-test nonsymbolic abilities, controlling for baseline nonsymbolic performance and condition.

Results are summarized in Table 2 (upper part). The model testing the refinement pathway from SNS to ANS accounted for approximately 63% of the variance (*R*^2^ = 0.63). Results revealed that pre-test symbolic abilities significantly predicted post-test nonsymbolic abilities (*B* = 0.164; *p* = 0.011), and the effect size was small (*β =* 0.227), as measured by the standardized coefficient. The baseline developmental effect of the nonsymbolic abilities from pre- to post-test was significant and large (*B =* 0.52; *p* < 0.001, *β* = 0.61). The condition effect was not *significant* (*p* = 0.217). Taken together, these results support H3a.

**Table 2:** Regression models testing developmental pathways between symbolic and nonsymbolic numerical abilities.

Model	Dependent variable	Predictor/effect	*B*	*p*-value	*β*
SNS → ANS *R*^2^ = 0.63	Post-test nonsymbolic	Pre-test symbolic	0.164	0.011	0.227
		Pre-test nonsymbolic, baseline	0.520	< 0.001	0.610
		Condition	—	0.217	—
ANS → SNS *R*^2^ = 0.67	Post-test symbolic	Pre-test nonsymbolic	0.298	0.004	0.243
		Pre-test symbolic, baseline	0.626	< 0.001	0.606
		Condition	3.491	0.001	0.204

SNS, symbolic numerical system; ANS, approximate number system. *β* refers to standardized regression coefficients. *R*^2^ refers to the variance explained by each full regression model. Baseline predictors represent within-system prediction from pre-test to post-test. Condition represents the adjusted difference between the experimental and control groups in post-test performance.

These findings suggest that children with stronger initial symbolic skills tended to show stronger later nonsymbolic performance, even after accounting for their initial nonsymbolic performance and group assignment. This is consistent with the predicted refinement pathway from SNS to ANS. Our findings showed that group membership did not significantly explain additional variance in post-test nonsymbolic performance, suggesting that the symbolic-to-nonsymbolic predictive relation was not simply attributable to differences between the experimental and control groups.

#### 3.3.2 Mapping pathway

Our third hypothesis, second part (H3b), proposed that the experimental and control groups would show different developmental patterns in the progression from ANS to SNS. Specifically, we expected pre-test nonsymbolic abilities to predict post-test symbolic skills in the experimental group but not in the control group, suggesting that number-line training may be associated with a stronger pathway from nonsymbolic to symbolic representations.

To test this, a linear regression model examined whether pre-test nonsymbolic abilities predicted post-test symbolic abilities, controlling for baseline symbolic performance and condition membership. Results are summarized in Table 2 (bottom part). The model testing the mapping pathway from ANS to SNS accounted for 67% of the variance (*R*^2^ = 0.669). Results indicated that pre-test nonsymbolic abilities significantly predicted post-test symbolic abilities (*B* = 0.298, *p* = 0.004), with a small-to-moderate effect size (*β =* 0.243), as measured by the standardized coefficient. The baseline developmental effect of symbolic abilities from pre- to post-test was also significant with a moderate effect size (*B* = 0.626, *p* < 0.001, *β* = 0.606). Finally, the condition effect was significant (*B* = 3.491, *p* = 0.001), with a small-to-moderate effect size (*β* = 0.204), as measured by the standardized coefficient.

These findings suggest that children with stronger initial nonsymbolic skills tended to show stronger later symbolic performance, even after accounting for their initial symbolic performance and group assignment. This is consistent with the predicted mapping pathway from ANS to SNS. Our findings showed that group membership explained additional variance in post-test symbolic performance, as measured by the small-to-moderate condition effect. This condition effect indicates an adjusted group difference in post-test symbolic performance. To test whether the mapping pathway differs between the experimental and control groups, *post-hoc* analyses were conducted.

#### 3.3.3 Mapping pathway across groups

In H3b, to formally test whether the developmental pathway from nonsymbolic pre-test to symbolic post-test performance $\left(\Delta_{\mathrm{NS}}=\beta_{\mathrm{NS},\text{experimental}}-\beta_{\mathrm{NS},\text{control}}\right)$ differed significantly between conditions, we conducted a full-sample interaction analysis using ANCOVA with interactions. The model showed a positive coefficient in the expected direction (*β* = 0.270) for the critical interaction term ( $NS_{\mathrm{pr}e_{z}}\times \text{Condition}$ ), although it did not reach standard statistical significance under conventional two-sided criteria (*p* = 0.090). The implied slopes were larger in the experimental group (0.332) than in the control group (0.063), suggesting a descriptively stronger nonsymbolic-to-symbolic association in the experimental group.

As a follow-up, we conducted a complementary Bayesian prior-sensitivity analysis to quantify the directional evidence for the slope difference between groups. Across the four prior specifications, the directional prediction remained highly stable and received between 92 and 96% posterior support; that is, the posterior probability that the nonsymbolic-to-symbolic slope was steeper in the experimental group than in the control group $\left(\Delta_{\mathrm{NS}}>0\right)$ ranged from 0.92 to 0.96. The 95% Crl included zero, indicating uncertainty regarding the exact magnitude of the slope difference. However, the posterior mass was consistently concentrated above zero, as indicated by directional posterior odds between 12:1 and 22:1 in favor of our predictions. Overall, these results indicate prior-stable, moderate directional support for a stronger nonsymbolic-to-symbolic relation in the experimental than the control group.

**Figure 4:**
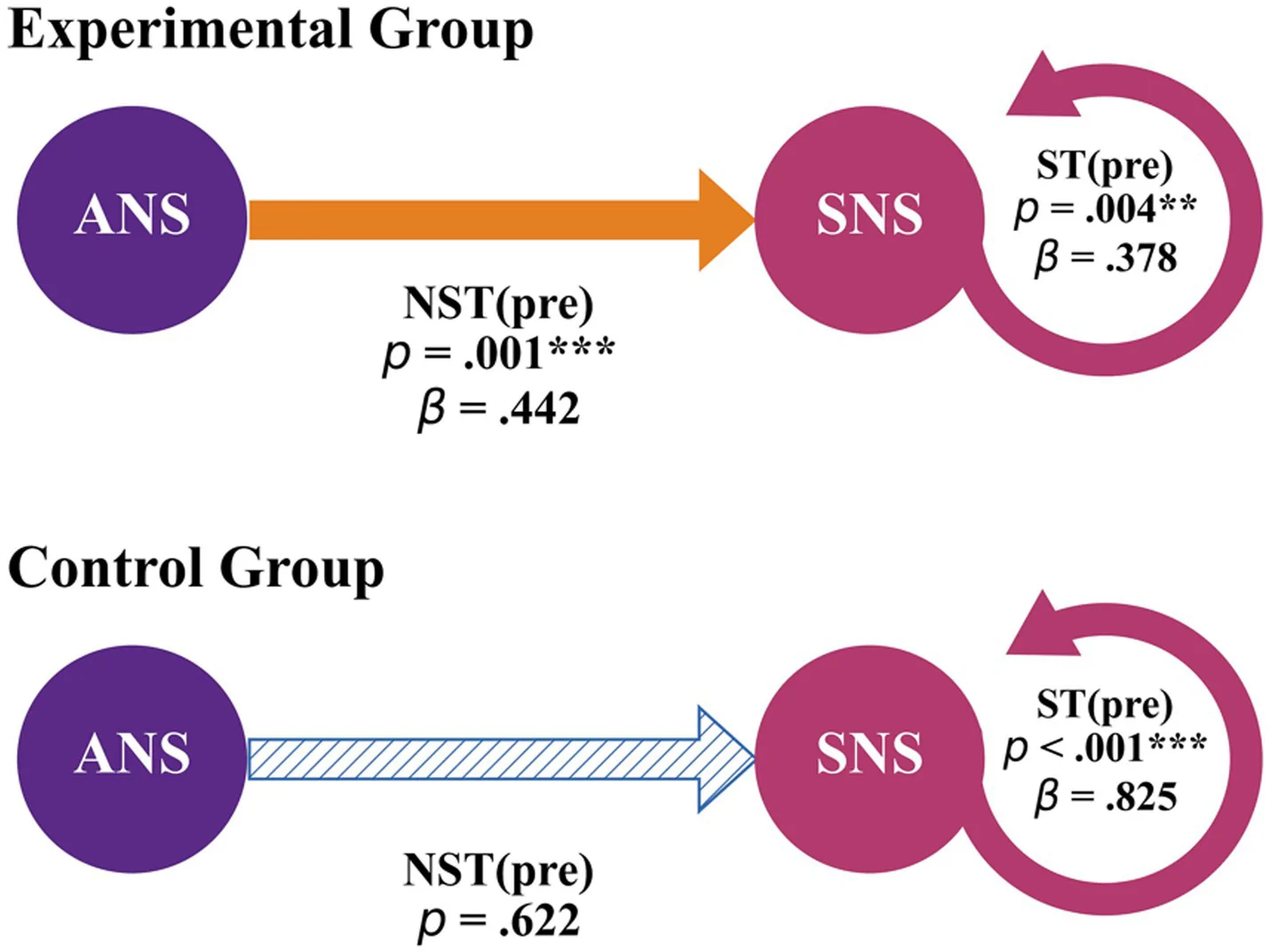
Developmental pathway from the approximate number system (ANS) to the symbolic numerical system (SNS), shown separately for the experimental and control groups. ANS is displayed on the left in purple, and SNS is displayed on the right in pink. The experimental group pathway is shown in orange with a solid straight arrow, and the control group pathway is shown in blue with a dashed straight arrow. Straight arrows represent the effect of pre-test nonsymbolic abilities (NST_pre) on post-test symbolic abilities, controlling for pre-test symbolic abilities. The solid orange arrow indicates a significant ANS-to-SNS pathway, whereas the dashed blue arrow indicates a nonsignificant pathway. Circular arrows represent the baseline effect of pre-test symbolic abilities (ST_pre) on post-test symbolic abilities within each group. Reported values show *p*-values and standardized regression coefficients (*β*).

Additionally, separate regressions were run for the experimental and control groups. The model testing mapping pathways in the experimental group explained 52% of the variance, as indicated by *R*^2^ = 0.52. Results are illustrated in the upper panel of Figure 4. The model revealed that pre-test nonsymbolic abilities significantly predicted post-test symbolic abilities (*B* = 0.408, *p* = 0.001), with a moderate effect size (*β* = 0.442). The baseline effect of symbolic abilities from pre- to post-test was significant (*B* = 0.314, *p* = 0.004), with a moderate effect size (*β* = 0.378), as measured with the standardized coefficient and illustrated in Figure 4 with circular arrows in the upper panel.

Finally, the model testing mapping pathways in the control group explained 76% of the variance (*R*^2^ = 0.76) and the results are illustrated in the bottom panel of Figure 4. The model showed that, for the control group, pre-test nonsymbolic abilities did not predict post-test symbolic abilities (*p* = 0.622). The baseline developmental effect of symbolic abilities from pre- to post-test was significant, with a large effect size (*B* = 0.906, *p* < 0.001, *β* = 0.825), as measured with the standardized coefficient and illustrated in Figure 4 with circular arrows in the bottom panel.

In sum, *post-hoc* results are consistent with the predicted different developmental patterns in the progression from ANS to SNS across conditions. The expanded interaction model showed that the interaction between condition and pre-test nonsymbolic abilities was positive and in the predicted direction, indicating a larger estimated slope in the experimental group than in the control group, although the two-sided test did not reach conventional significance. Bayesian sensitivity analysis showed that the directional prediction remained highly stable and received between 92 and 96% posterior support, consistent with the interpretation that the mapping pathway may have been stronger in the experimental group. Finally, the group-specific simple-slope analyses illustrated the same pattern: the relation from pre-test nonsymbolic to post-test symbolic was significant in the experimental group, whereas the corresponding association was weak and nonsignificant in the control group. Taken together, the frequentist interaction, Bayesian sensitivity analyses, and group-specific regressions yielded converging, moderate directional evidence that the mapping-related predictive relation was stronger in the experimental group. This pattern is consistent with an association between number-line training and a stronger approximate-to-symbolic predictive relation, in line with H3b.

Finally, exploratory sensitivity analyses including sex as an additional covariate did not materially alter the interpretation of the primary findings, as summarized in Supplementary Table S2.1. The sex coefficient was not statistically significant in the model testing the direct effect of number-line training on number-line estimation abilities (H1; *B* = 0.145, *p* = 0.136) or in the model testing the transfer effect of training on early mathematics abilities (H2; *B* = −0.043, *p* = 0.352). Similarly, the sex coefficient was not statistically significant in the model testing the SNS-to-ANS relation (H3a; *B* = 0.622, *p* = 0.422) or in the model testing the ANS-to-SNS relation (H3b; *B* = 1.996, *p* = 0.053). Additionally, the Session × Condition effects remained comparable in the NLET model (*B* = −0.364, *p* = 0.043) and the ABMT model (*B* = 0.079, *p* = 0.020). Similarly, the focal predictive relations remained statistically significant in the H3 models after adjustment for sex, both for the refinement pathway from pre-test symbolic to post-test nonsymbolic performance (*B* = 0.169, *p* = 0.009) and for the mapping pathway from pre-test nonsymbolic to post-test symbolic performance (*B* = 0.307, *p* = 0.003).

## 4 Discussion

In this study, we examined whether practicing number-line estimation through a brief, game-based intervention enhances preschoolers’ number-line accuracy and early math abilities (Objective 1), and whether such training is associated with the developmental dynamics between symbolic and nonsymbolic numerical systems (Objective 2). To address these aims, we employed a 2 × 2 pre–post design with experimental and control groups. The experimental group received three training sessions using the frog-game task, in which children estimated the location of numbers on a 0–20 number-line and received trial-by-trial feedback.

This design allowed us to distinguish developmental changes associated with regular preschool exposure from those associated with the targeted training. By comparing pre–post changes across groups while also examining predictive relations between symbolic and nonsymbolic abilities, the study aims to contribute to the understanding of both number-line intervention effects and short-term developmental dynamics in numerical cognition.

Overall, our study brings together perspectives from developmental numerical cognition and educational psychology to examine how number-line training may support early mathematical development while being associated with the interaction between symbolic and nonsymbolic systems. Below, we first discuss the effectiveness of the intervention and its transfer effect on early math abilities (Section 4.1). We then examine the developmental dynamics between symbolic and nonsymbolic processing and the role of number-line training in these pathways (Section 4.2). Finally, we outline limitations and directions for future research (Section 4.3) and draw conclusions for our study (Section 4.4).

### 4.1 Intervention effect on number-line and early math abilities

Our results indicated that number-line skills improved with the intervention, with a large effect. These findings are consistent with prior intervention studies reporting improvements in estimation accuracy following number-line training (Ramani and Siegler, 2011; Siegler and Ramani, 2009; Ramani and Siegler, 2008; Ramani et al., 2012; Honoré and Noël, 2016). For instance, Honoré and Noël (2016) reported similar improvements in number-line abilities following training in symbolic or nonsymbolic magnitude comparison and number-line estimation. In their study, preschoolers underwent a 10-session training program, with significant gains observed in number-line estimation tasks. In contrast, our intervention consisted of only three sessions and focused exclusively on number-line estimation. Despite the shorter training period, our results showed a significantly larger reduction in estimation error in the experimental group compared to the control group. These findings suggest that even brief, targeted training can produce meaningful improvements in number-line estimation abilities.

Beyond the direct effect on estimation accuracy, our findings also indicate a modest but significant transfer effect to early math abilities. The experimental group showed greater gains in ABMT performance than the control group, corresponding to an approximately 8% relative improvement. This finding is consistent with previous intervention studies reporting a transfer effect from number-line training to broader math abilities (Ramani and Siegler, 2011; Siegler and Ramani, 2009; Kucian et al., 2011; Maertens et al., 2016; Honoré and Noël, 2016; Mera et al., 2022). For example, Siegler and Ramani (2009) observed a substantial reduction in arithmetic error following experience with a linear number board, whereas Maertens et al. (2016) reported a medium improvement in pictorial arithmetic following number-line estimation training. Honoré and Noël (2016) found a large effect on exact addition after symbolic magnitude training that included number-line activities, while Mera et al. (2022), using a broader intervention that combined number-line estimation with other numerical activities, reported very large pre–post gains in general mathematical competence in both intervention groups. In this context, the transfer observed in the present study is modest but practically meaningful, particularly because it emerged after only three brief sessions focused specifically on number-line estimation. From an educational perspective, this suggests that short, game-based number-line activities may provide a useful and time-efficient complement to broader preschool mathematics instruction by producing benefits that extend beyond the trained estimation task.

Our results contrast with other studies where no transfer effect was identified (e.g., Lunardon et al., 2023; Wei et al., 2023). One possible reason for a positive transfer in our case lies in the specific training format. The frog-game used in the intervention was based on the classical bounded number-line estimation paradigm, where children are presented with a blank line labeled only at the endpoints, allowing for the application of either counting-based or proportional judgment strategies. Although the target 10 (midpoint) was overrepresented in the target number list—drawing participants’ attention to the benefit of using it as a reference for estimating nearby targets—there was no mark in the middle of the line. This design intentionally encouraged a flexible application of estimation strategies. Other training designs, such as those using “The Number Line” game in Lunardon et al. (2023), included intermediate tick marks or numbers, which may reduce estimation demands and encourage reliance on specific strategies rather than flexible estimation. This variation in task design may help explain the differing results observed across interventions, as the effectiveness of training may depend on how its format aligns with the cognitive demands of the estimation task, which can influence the degree of improvement and transfer to other mathematical abilities.

Overall, with respect to Objective 1, the present findings provide evidence that brief number-line estimation training improved estimation accuracy and early mathematical performance. The results contribute to the literature by suggesting that transfer effects may depend on task design features that encourage flexible and adaptive use of estimation strategies.

### 4.2 Symbolic and nonsymbolic numerical processing: developmental dynamics

The second objective of our study was to examine how symbolic and nonsymbolic numerical systems interact over a short time window and whether number-line training is associated with differences in the developmental dynamics between them, in relation to predictions from the mapping (ANS-to-SNS) and refinement (SNS-to-ANS) accounts. Results revealed a bidirectional relation between symbolic and nonsymbolic skills, providing support for both refinement and mapping accounts. Specifically, pre-test symbolic abilities predicted post-test nonsymbolic performance, consistent with the refinement account (Matejko and Ansari, 2016; Lyons et al., 2018; Lau et al., 2021; Hutchison et al., 2020; Mou et al., 2023), whereas pre-test nonsymbolic abilities predicted post-test symbolic performance, consistent with the mapping account (Dehaene and Changeux, 1993; Feigenson et al., 2004; Piazza, 2010; Szkudlarek and Brannon, 2017; Park and Brannon, 2013, 2014; Hyde et al., 2014). In addition, the analyses of developmental dynamics showed that the mapping pathway from ANS to SNS was associated with the number-line training, as indicated by *post-hoc* analyses that yielded moderate directional evidence that the mapping-related predictive relation was stronger in the experimental group, consistent with an association with number-line training.

Regarding the refinement account, our findings align with previous longitudinal studies showing that symbolic numerical skills predict subsequent nonsymbolic numerical development (Matejko and Ansari, 2016; Lyons et al., 2018; Lau et al., 2021; Mou et al., 2023). Mou et al. (2023) showed that verbal number knowledge predicted later ANS precision both over a brief two-week interval and over a 1-year interval, with stronger evidence across the longer developmental window. Similarly, other evidence in favor of the refinement account has come from longitudinal studies examining symbolic-to-nonsymbolic relations across relatively broad developmental windows, including the first year of formal schooling in Matejko and Ansari (2016), the kindergarten school year in Lyons et al. (2018), and three time points spanning kindergarten to Grade 1 in Lau et al. (2021). In line with this background, the small effect size observed in the present study should be interpreted in relation to the shorter temporal scale of the design. The present findings do not necessarily indicate that the refinement pathway is weak; rather, they suggest that this developmental dynamic can be detected even over a brief 5–7-week interval, although its magnitude may be more limited than effects estimated over broader developmental windows. Thus, alongside the short-interval analysis reported by Mou et al. (2023), the present study contributes to this literature by extending evidence for the refinement pathway to a short-term pre–post design.

Regarding the mapping perspective, our findings contribute to the debate about whether approximate magnitude abilities provide a foundation for symbolic numerical development (Szkudlarek and Brannon, 2017; Qiu et al., 2021; Lau et al., 2021; Libertus, 2019; Odic and Starr, 2018; Szűcs and Myers, 2017). As reviewed in the introduction, the mapping account is supported by evidence showing associations between ANS acuity and mathematical achievement, as well as by intervention studies in which nonsymbolic approximate arithmetic training improved symbolic math performance (Chen and Li, 2014; Fazio et al., 2014; Schneider et al., 2017; Hyde et al., 2014; Park and Brannon, 2013, 2014). However, this literature remains mixed. Longitudinal studies have not found evidence that nonsymbolic skills predict later symbolic development under regular educational exposure (Matejko and Ansari, 2016; Lyons et al., 2018; Lau et al., 2021), and meta-analytic and critical reviews of ANS training studies have questioned whether training approximate magnitude processing alone produces reliable transfer to symbolic mathematical outcomes (Szűcs and Myers, 2017; Qiu et al., 2021). In this context, the converging pattern observed in the *post-hoc* analyses suggests that the mapping-related predictive relation was stronger in the experimental group than in the control group. This finding points to the possibility that the ANS-to-SNS dynamic is more likely to emerge when children are supported in coordinating nonsymbolic numerical information with symbolic representations. This interpretation is consistent with prior work showing that number-line estimation is linked to both approximate and exact numerical abilities (Gunderson and Hildebrand, 2021), and with longitudinal evidence that number-line accuracy may mediate the relation between ANS acuity and symbolic arithmetic skills (Wong et al., 2016). Thus, mixed findings in the broader mapping-account literature may partly reflect variability in whether children have developed, or are experimentally supported to use, number-line estimation abilities that integrate approximate magnitudes with symbolic targets. The frog-game training may have supported the mapping pathway by requiring children to integrate approximate numerical estimation with symbolic target interpretation in a bounded number-line context lacking intermediate anchors and providing feedback. The absence of explicit reference points encouraged children to flexibly apply estimation strategies while trial-by-trial feedback may have reinforced successful mappings between approximate magnitude representations and symbolic outputs.

Overall, in relation to the second objective, our findings provide evidence in favor of both the refinement and mapping accounts and suggest that brief number-line training can be associated with short-term relations between numerical systems, with both theoretical and practical implications. From a theoretical perspective, our findings suggest bidirectionality in the interaction between ANS and SNS, which may follow different developmental dynamics depending on whether preschool children are involved in regular school activities or are exposed to additional targeted number-line training, helping to explain mixed findings on the study of the development of numerical systems.

From a practical perspective, our findings highlight the potential of training based on number-line estimation as a developmentally sensitive and practical tool to support symbolic numerical learning. By drawing on the intuitive approximate abilities that children already possess, this type of intervention may support symbolic understanding by engaging accessible processes that connect approximate and symbolic representations. This aligns with established principles of instructional design (Gulz and Haake, 2024; Laurillard, 2016), emphasizing the importance of well-designed feedback and scaffolding. This suggests a promising direction for designing educational strategies that activate and connect existing numerical systems via game-like experiences that are easy to implement and scalable across educational settings.

### 4.3 Limitations and suggestions for future research

One limitation concerns the absence of long-term follow-up. In the Swedish educational context, ethical considerations favored providing beneficial learning opportunities to all children. After the post-test session, children in the control group were therefore offered the frog-game training. As a result, the control group could no longer serve as a comparison group for assessing long-term intervention effects. The lack of follow-up limits our ability to examine whether the refinement pathway from symbolic to approximate magnitude processing changes over a longer developmental window. In the present study, the refinement-related predictive relation emerged with a small effect size after controlling for condition. A longer follow-up could reveal whether the strength of this relation changes across broader developmental intervals or differs across learning contexts.

A second limitation concerns the short duration of the intervention. Although the brevity of the training can be considered a strength from an applied perspective, as it suggests that meaningful improvements in number-line estimation can be achieved with limited instructional time, it may also have limited the extent of transfer to broader mathematical abilities. In the present study, the transfer effect to early math performance was significant but modest, suggesting that a longer period of practice may be necessary for gains in number-line estimation to generalize more strongly to other mathematical domains. Future studies should examine whether more extended number-line estimation training, particularly interventions based on bounded number lines without intermediate marks and incorporating trial-by-trial feedback, can produce larger transfer effects on early math abilities.

A third limitation concerns the classroom-based structure of the study. Randomization was conducted primarily at the classroom level, with one classroom randomly split at the individual-child level to maintain comparable group sizes. With children nested within classrooms and schools, this may limit the generalization of the results. However, this procedure allowed the intervention to be implemented within the natural preschool context, minimizing disruption to ordinary classroom routines and reducing the risk that some children within the same classroom would feel excluded from the game-based training. Future studies including a larger number of classrooms could extend the present findings by explicitly modeling classroom- and school-level variation and examining whether the observed intervention effects remain stable across educational settings.

A fourth limitation concerns the study’s focus on group-level effects and its limited capacity to examine individual differences. The use of GEE was appropriate for testing population-averaged changes between the experimental and control groups, but it did not allow a detailed examination of heterogeneity in intervention response. In addition, exact chronological age was not collected in accordance with the data-minimization requirements of the approved ethical protocol, preventing us from examining age-related variability within the sample. Future studies using larger samples, more precise age information, and subject-specific modeling approaches could investigate interindividual variation in developmental change in relation to number-line training, thereby enabling a more fine-grained examination of how age and other individual characteristics influence the relations among number-line estimation, symbolic and nonsymbolic numerical skills, and broader mathematical competence.

Finally, further research is needed to investigate the cognitive mechanisms underlying number-line estimation as a bridge between numerical systems. Computational approaches, such as Drift Diffusion Modeling, may help clarify the decision processes involved in estimation and how training supports integration between approximate and symbolic representations.

### 4.4 Conclusion

In sum, brief number-line estimation training enhanced preschoolers’ estimation accuracy and early math abilities. The findings also revealed bidirectionality in the predictive relations between symbolic and nonsymbolic numerical systems, providing support for both refinement and mapping accounts. Symbolic abilities predicted later nonsymbolic performance after controlling for baseline nonsymbolic performance and condition, whereas nonsymbolic abilities predicted later symbolic performance after controlling for baseline symbolic performance and condition. Importantly, converging *post-hoc* analyses provided moderate directional evidence that the approximate-to-symbolic predictive relation was stronger in the experimental group than in the control group, consistent with an association between number-line training and enhanced coordination between numerical systems. Together, these findings position number-line estimation as both an effective educational activity and a potential bridge between approximate and symbolic numerical representations. Limitations regarding the absence of long-term follow-up, classroom-based randomization and the brief training invite future research to examine these relations under new circumstances.

## Data Availability

The original contributions presented in the study are included in the article/Supplementary material, further inquiries can be directed to the corresponding author.

## Footnotes

1
https://pavlovia.org
