# Supplementary material for: Number-line estimation training in preschoolers supports early mathematical development and is associated with a stronger approximate-to-symbolic numerical pathway
Source: Front Psychol. 2026 Sep 4;17:1883264. doi: 10.3389/fpsyg.2026.1883264 (PMC13585542; doi:10.3389/fpsyg.2026.1883264)
Supplement: Supplementary file 1 [file Data_Sheet_1.PDF]

Supplementary Material 1. Bayesian prior-sensitivity analysis of the nonsymbolic-to-symbolic slope difference

1. Purpose and model specification

The second part of Hypothesis 3 (H3b) proposed that the experimental and control groups would show different developmental patterns in the progression from approximate magnitude processing to symbolic numerical performance. Specifically, H3b predicted that pre-test nonsymbolic abilities would predict post-test symbolic skills in the experimental group, but not in the control group, suggesting that number-line estimation training may be associated with the nonsymbolic-to-symbolic mapping pathway.

The difference between experimental and control groups in the pathway from nonsymbolic to symbolic abilities, as evaluated by the slope differences between conditions, was first examined using a full-sample interaction model testing whether the predictive relation from pre-test nonsymbolic abilities to post-test symbolic skills differed between conditions. The model indicated that the focal interaction term, NS_pre_z × Condition, was positive and in the expected direction, indicating a larger estimated slope in the experimental group than in the control group, although the two-sided frequentist test did not reach conventional statistical significance, $\beta = 0.270$, $p = .090$. The implied simple slopes were 0.332 in the experimental group and 0.063 in the control group. Follow-up group-specific regressions showed the same descriptive pattern: NS_pre significantly predicted S_post in the experimental group, $B = 0.408$, $p = .001$, whereas the corresponding association was not significant in the control group, $p = .622$.

The present supplementary analysis complements these results by estimating the posterior distribution of the slope difference between the experimental and control groups. This analysis was included because the pattern implied by H3b is directional: the NS_pre → S_post slope was expected to be larger in the experimental group than in the control group. The Bayesian analysis therefore quantifies the directional evidence for this slope difference, rather than treating the group-specific regressions as separate tests of the same question.

The focal parameter was:

$$\Delta_{NS} = \beta_{NS,experimental} - \beta_{NS,control}$$

where Δ_{NS} represents the difference between the nonsymbolic-to-symbolic predictive slope in the experimental group and the corresponding slope in the control group. Positive values of Δ_{NS} therefore indicate a stronger NS_pre → S_post relation in the experimental group.

The Bayesian model used the same structure as the frequentist interaction model reported in the main text:

$$\begin{aligned} S\_post\_z \sim\ & S\_pre\_z + NS\_pre\_z + Condition + S\_pre\_z \times Condition \\ & + NS\_pre\_z \times Condition \end{aligned}$$

The dependent variable was standardized post-test symbolic performance. Predictors included standardized pre-test symbolic performance, standardized pre-test nonsymbolic performance, condition, and the interactions between condition and both pre-test predictors. The focal term was the NS_pre_z × Condition interaction, which directly estimates Δ_{NS}.

The analysis included the same participants as the frequentist interaction model: $N = 98$, with 52 children in the control group and 46 in the experimental group. All continuous variables were standardized before modeling. Participants were included when they had complete symbolic and nonsymbolic adjusted scores at both pre-test and post-test.

Because all continuous variables were standardized, the primary prior for the focal interaction term was specified as a weakly informative prior centered on zero:

$$NS\_pre\_z \times Condition \sim Normal(0,0.25)$$

This prior reflects the expectation that the slope difference, if present, would likely be small to moderate rather than extremely large. To evaluate the robustness of the directional pattern to prior specification, three additional priors were examined: $Normal(0,0.50)$, $Normal(0,1.00)$, and the Bambi default prior. The Bambi default prior was treated as a broad sensitivity case rather than as the preferred prior, because its scale is very wide for a standardized interaction coefficient.

Only the prior on the focal NS_pre_z × Condition interaction was varied across models. The model structure was otherwise identical across all prior specifications.

MCMC sampling used four chains, 1000 tuning iterations, and 2000 posterior draws per chain, with target_accept = 0.90 and random_seed = 42. Convergence diagnostics indicated satisfactory sampling, with maximum $\hat{R} = 1.00$ across models.

The main Bayesian quantity of interest was $P(\Delta_{NS} > 0)$, which estimates the posterior probability that the nonsymbolic-to-symbolic slope was larger in the experimental group than in the control group. We also report directional posterior odds, which express directional

evidence as the ratio of posterior mass above zero to posterior mass below zero, with larger odds indicating stronger posterior support for a positive slope difference. Finally, uncertainty in the estimated magnitude of Δ_{NS} was summarized using 95% credible intervals, computed as highest-density intervals (HDIs), which contain the most credible 95% of the posterior distribution.

2. Results

Results are summarized in Table S1. Across all prior specifications, the posterior mean of Δ_{NS} was positive, ranging from 0.19 to 0.27 standardized units. The posterior probability that Δ_{NS} was greater than zero was stable across priors, ranging from .92 to .96. This corresponds to directional posterior odds ranging from approximately 12:1 to 22:1 in favor of a larger nonsymbolic-to-symbolic slope in the experimental group.

Table S1: Bayesian prior-sensitivity results for the nonsymbolic-to-symbolic slope difference.

Prior on NS_pre_z × Condition	Prior SD	Mean Δ_{NS}	95% CrI (HDI)	P ($\Delta_{NS} > 0$)	Directional odds	Max $\hat{R}$
**Normal (0, 0.25)**	0.25	0.189	[-0.065, 0.466]	.920	11.5:1	1.00
**Normal (0, 0.50)**	0.50	0.242	[-0.054, 0.549]	.939	15.4:1	1.00
**Normal (0, 1.00)**	1.00	0.266	[-0.052, 0.570]	.956	21.7:1	1.00
**Bambi default**	3.947	0.267	[-0.063, 0.582]	.947	17.8:1	1.00

Note. Δ_{NS} represents the difference between the NS_pre → S_post slope in the experimental group and the corresponding slope in the control group. Positive values indicate a larger slope in the experimental group. CrI = credible interval, computed as a highest-density interval (HDI).

These results are illustrated in Figure S1, which shows the posterior distributions of Δ_{NS}under the primary weakly informative prior $Normal(0,0.25)$(blue), the broader sensitivity priors $Normal(0,0.50)$(orange) and $Normal(0,1.00)$(green), and the Bambi default prior (red). Across all prior specifications, the posterior distributions were shifted in the predicted positive direction, as indicated by the concentration of most posterior mass to the right of zero. Under the primary weakly informative prior, the posterior mean of $\Delta_{NS}$was 0.189 standardized units, with $P(\Delta_{NS} > 0) = .920$. Broader priors produced slightly larger posterior means, but the directional probability remained stable.

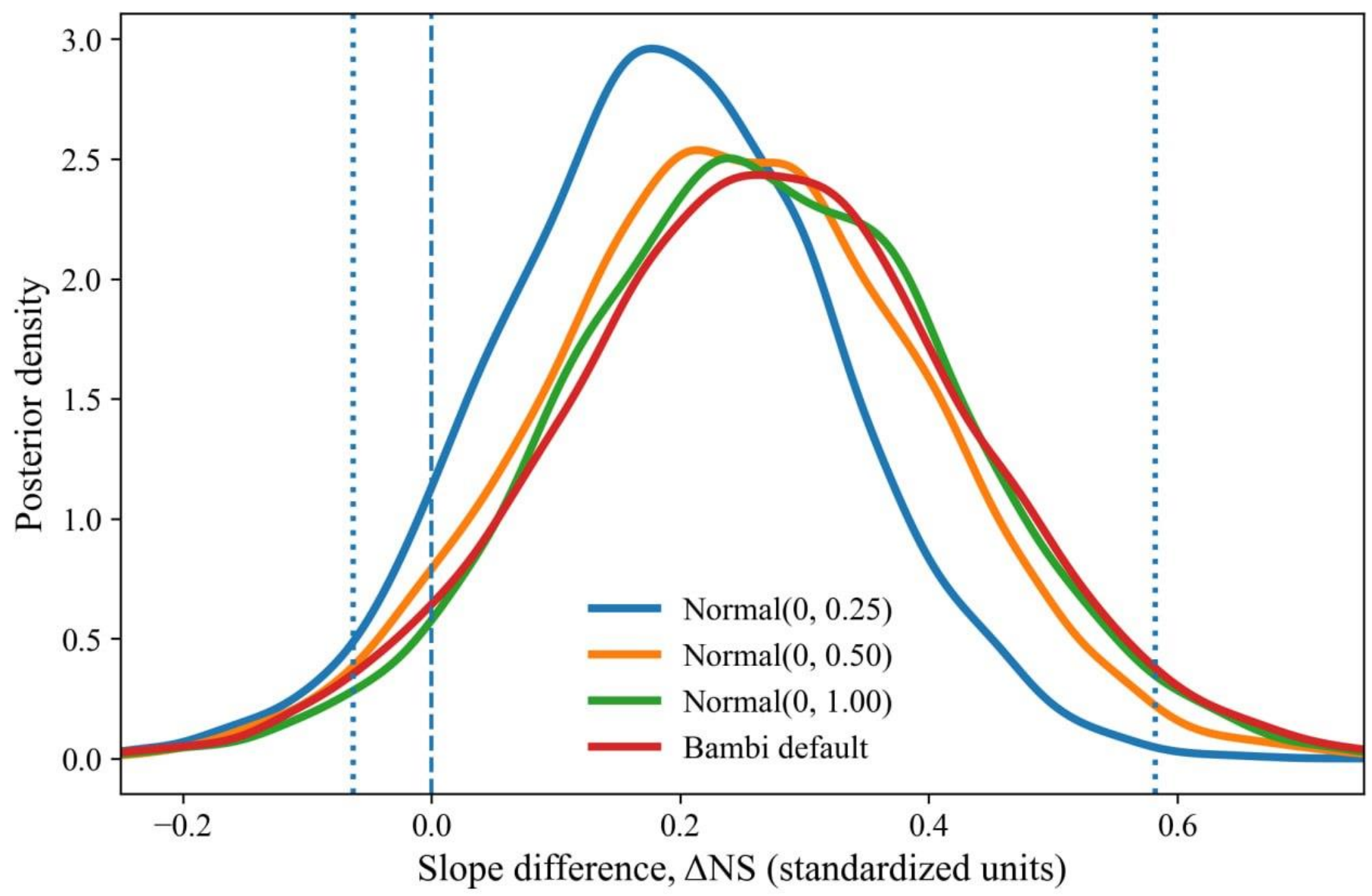

Figure S1: Posterior distributions represent $\Delta_{NS} = \beta_{NS,experimental} - \beta_{NS,control}$, the difference between the NS_pre → S_post slope in the experimental and control groups. The blue curve corresponds to the primary weakly informative prior $Normal(0,0.25)$, the orange curve to $Normal(0,0.50)$, the green curve to $Normal(0,1.00)$, and the red curve to the Bambi default prior. The dashed vertical line marks zero, corresponding to no slope difference between conditions. Dotted vertical lines indicate the 95% credible interval, computed as the highest-density interval, for the Bambi default prior.

The 95% credible intervals, computed as highest-density intervals, included zero under all prior specifications, indicating uncertainty regarding the exact magnitude of the slope difference. However, the posterior mass was consistently concentrated above zero, indicating that the data were more compatible with a positive than with a negative slope difference.

3. Interpretation

This Bayesian prior-sensitivity analysis supports the condition-related pattern in the ANS-to-SNS pathway. Across all priors, the estimated slope difference was positive, and the posterior probability that the nonsymbolic-to-symbolic slope was larger in the experimental group ranged from .92 to .96. This suggests that the observed pattern was not dependent on a single prior specification and that the directional prediction received between 92% and 96% posterior support.

At the same time, the credible intervals included zero, indicating that the magnitude of the slope difference remains uncertain. Therefore, the Bayesian results should not be interpreted as definitive proof that the intervention causally altered an underlying developmental mechanism. Rather, they indicate that the data consistently favored the direction expected, with a stronger NS_pre → S_post predictive relation in the experimental group than in the control group.

Taken together with the frequentist interaction model and the group-specific simple-slope analyses reported in the main text, this analysis provides converging directional support for the interpretation that number-line estimation training was associated with a stronger nonsymbolic-to-symbolic predictive relation.
