# Supplementary material for: Number-line estimation training in preschoolers supports early mathematical development and is associated with a stronger approximate-to-symbolic numerical pathway
Source: Front Psychol. 2026 Sep 4;17:1883264. doi: 10.3389/fpsyg.2026.1883264 (PMC13585542; doi:10.3389/fpsyg.2026.1883264)
Supplement: Supplementary file 2 [file Data_Sheet_2.PDF]

Supplementary Material 2. Exploratory sensitivity analyses including sex and baseline mathematical competence as covariates

1. Purpose and scope

The main analyses reported in the manuscript focused on number-line estimation training effects and on predictive relations between symbolic and nonsymbolic numerical skills. The present supplementary analyses provide exploratory sensitivity checks examining whether the main findings remain stable when sex is included as an additional covariate.

These analyses address two complementary questions. First, we examined whether adding sex to the primary models changed the focal effects reported in the manuscript. Second, for the H3 regression models, we examined whether sex and broader baseline mathematical competence indicators contributed to post-test symbolic and nonsymbolic performance when included together with the original predictors.

Broader baseline mathematical competence was operationalized using pre-test Ani Banani Math Test (ABMT) scores and pre-test Number Line Estimation Task (NLET) PAE scores. The term sex is used because the available variable classified children as female or male; gender identity was not assessed.

2. Analytic samples and model specification

The H1 and H2 sex-adjusted GEE models were estimated using the final analytic sample of 114 participants. The H3 models adjusted for sex used the complete H3 sample of 98 participants. Because two participants had missing pre-test PAE scores, the H3 models

additionally adjusted for broader baseline mathematical competence were estimated using a listwise-complete sample of 96 participants.

For H1 and H2, the primary generalized estimating equation (GEE) models were re-estimated with sex added as an exploratory covariate. The model structure was otherwise the same as in the main analyses, using a Gamma distribution with log link and participant ID as the clustering variable:

$$PAE \sim Session \times Condition + Sex$$

$$ABMT \sim Session \times Condition + Sex$$

For H3, two complementary analyses were conducted. First, the two primary full-sample linear regression models (H3a and H3b) were also re-estimated with sex added as an additional covariate:

$$NS_{post} \sim NS_{pre} + S_{pre} + Condition + Sex$$

$$S_{post} \sim NS_{pre} + S_{pre} + Condition + Sex$$

In the H3a model, the focal coefficient was the relation between pre-test symbolic performance and post-test nonsymbolic performance, after adjustment for baseline nonsymbolic performance, condition, and sex. In the H3b model, the focal coefficient was the relation between pre-test nonsymbolic performance and post-test symbolic performance, after adjustment for baseline symbolic performance, condition, and sex.

Second, the two primary H3 models were re-estimated with sex, pre-test PAE, and pre-test ABMT performance included as additional covariates:

$$NS_{post} \sim NS_{pre} + S_{pre} + Condition + Sex + PAE_{pre} + \ ABMT_{pre}$$

$$S_{post} \sim NS_{pre} + S_{pre} + Condition + Sex + PAE_{pre} + \ ABMT_{pre}$$

These models examined whether the focal predictive relations underlying the refinement and mapping pathways remained statistically significant after adjustment for sex and complementary indicators of broader baseline mathematical competence. They also examined whether pre-test PAE and pre-test ABMT performance accounted for additional variance in symbolic and nonsymbolic post-test performance beyond the predictors included in the primary H3 models.

3. Results

3.1. Stability of the focal effects after adding sex

Adding sex as an exploratory covariate did not materially change the focal effects reported in the manuscript. The Session × Condition effects in the H1 and H2 GEE models remained comparable to those obtained in the primary analyses. Similarly, the focal predictive relations examined in the H3 models remained statistically significant after adjustment for sex.

In the H3a model, pre-test symbolic performance continued to predict post-test nonsymbolic performance after controlling for baseline nonsymbolic performance, condition, and sex. In the H3b model, pre-test nonsymbolic performance continued to predict post-test symbolic performance after controlling for baseline symbolic performance, condition, and sex.

The sex coefficient was not statistically significant in any of the four sex-adjusted models (H1: $B = 0.145$, $p = .136$; H2: $B = -0.043$, $p = .352$; H3a: $B = 0.622$, $p = .422$; H3b: $B = 1.996$, $p = .053$). These results are summarized in Table S2.1.

Table S2.1: Primary and sex-adjusted sensitivity models for the focal effects

Analysis	Outcome	Primary model: focal effect	Sex-adjusted sensitivity model: focal effect	Sex coefficient in adjust model
**H1 GEE**	PAE	Session × Condition: B = -0.388, p = .032	Session × Condition: B = -0.364, p = .043	B = 0.145, p = .136
**H2 GEE**	ABMT	Session × Condition: B = 0.079, p = .021	Session × Condition: B = 0.079, p = .020	B = −0.043, p = .352
**H3a regression**	NS_{post}	$S_{pre} \rightarrow NS_{post}$ B = 0.164, p = .011	$S_{pre} \rightarrow NS_{post}$ B = 0.169, p = .009	B = 0.622, p = .422
**H3b regression**	S_{post}	$NS_{pre} \rightarrow S_{post}$ B = 0.298, p = .004	$NS_{pre} \rightarrow S_{post}$ B = 0.307, p = .003	B = 1.996, p = .053

Note. The sex-adjusted sensitivity model uses the same specification as the corresponding primary model, with sex added as an additional covariate. Male was coded relative to female.

3.2. Covariate-adjusted H3 regression models

We next examined whether sex and broader baseline mathematical competence indicators contributed to the H3 regression models when included together.

Table S2.2: Covariate-adjusted H3 regression models

**Outcome**	**Predictor**	***B***	***p*-value**
S_{post}	S_{pre}	0.606	< .001
	NS_{pre}	0.256	.014
	Condition	3.294	.002
	Male	1.746	.096
	PAE_{pre}	0.087	.242
	$ABMT_{pre}$	0.527	.039
NS_{post}	S_{pre}	0.148	.038
	NS_{pre}	0.512	< .001
	Condition	0.688	.379

Male	0.260	.740
PAE_{pre}	0.051	.362
$ABMT_{pre}$	0.172	.367

Note. For the S_{post} model, R^2 = .696. For the SN_{post} model, R^2 = .633. The covariate-adjusted H3 models included the original H3 predictors plus sex, pre-test PAE, and pre-test ABMT performance. Male was coded relative to female. Condition was coded relative to the control group. S_{pre} and S_{post} denote symbolic pre- and post-test scores, respectively; NS_{pre} and NS_{post} denote nonsymbolic pre- and post-test scores.

For symbolic post-test performance, the covariate-adjusted model remained consistent with the primary H3b analysis. S_{pre}, NS_{pre}, and condition remained significant predictors. Sex was not a statistically significant covariate in the S_{post} model. $ABMT_{pre}$ was also associated with S_{post} in the covariate-adjusted model. For nonsymbolic post-test performance, S_{pre} and NS_{pre} remained significant predictors, whereas condition, sex, PAE_{pre}, and $ABMT_{pre}$ were not statistically significant. These results are summarized in Table S2.2.

4. Interpretation

Taken together, these exploratory sensitivity analyses indicate that including sex did not materially alter the interpretation of the main findings. When sex was added to the primary models, the focal effects for H1, H2, H3a, and H3b remained comparable to those reported in the manuscript.

The covariate-adjusted H3 models led to the same qualitative interpretation as the primary H3 analyses. The refinement pathway from symbolic to nonsymbolic performance remained significant, and condition did not explain additional variance in nonsymbolic post-test performance. The mapping-related prediction from nonsymbolic to symbolic performance also

remained significant, and condition continued to explain additional variance in symbolic post-test performance. Sex was not a statistically significant covariate in either covariate-adjusted H3 model.

Although sex was not a statistically significant covariate in either H3 model, the coefficient for sex in the H3b model approached the conventional .05 significance threshold. This finding should not be interpreted as evidence of a sex difference, particularly given the exploratory nature of the analysis and the available sample size. Nevertheless, it points to a potential sex-related pattern in symbolic post-test performance that needs further investigation. Future studies with larger samples and designs specifically developed to test moderation could examine this possibility more directly and determine whether sex moderates the predictive relation between nonsymbolic and symbolic numerical performance.

Finally, pre-test ABMT performance also showed a positive association with symbolic post-test performance. Given the exploratory nature of the analysis, the multiple covariates examined, and the conceptual overlap between broader early mathematical competence and symbolic numerical performance, this association should be interpreted cautiously. Future studies specifically designed to investigate this association could examine whether it varies as a function of individual differences and identify which components of broader mathematical competence account for it.

Overall, these analyses support the stability of the main findings under the covariate adjustments examined here.
